# Analysis of tuberculosis prevalence surveys in Africa: evidence to change the status quo in case finding

**DOI:** 10.3389/fpubh.2026.1941743

**Published:** 2026-09-24

**Authors:** Joia S. Mukherjee, Melino Ndayizigye, Carissa Lee, Matthew Gilmore, Sirine Benali, Llang Maama, Afom T. Andom

**Affiliations:** 1 Department of Medicine, Massachusetts General Brigham, Boston, MA, United States; 2 Partners In Health, Boston, MA, United States; 3 Chelsea and Westminster Hospital NHS Foundation Trust, London, United Kingdom; 4 Northeastern Bouve College of Health Sciences, Boston, MA, United States; 5 Ariadne Laboratory, Boston, MA, United States; 6 Government of Lesotho, Maseru, Lesotho; 7 Partners In Health, Maseru, Lesotho

**Keywords:** Africa, artificial intelligence, chest X-ray, GeneXpert, TB case finding, TB screening, tuberculosis prevalence survey

## Abstract

**Introduction:**

Lesotho has among the world’s highest TB prevalences and the national program finding only 50% of incident cases. Lesotho underwent its first TB prevalence study in 2019 in reviewing this study, the authors recognized that the standard measures for screening and diagnosis TB often failed to detect the presence of culture positive pulmonary TB. This review seeks to determine if the systematic under detection of culture positive TB by standard methods is true among prevalence surveys in Africa.

**Methods:**

We conducted a review to identify TB prevalence surveys conducted in Africa from 2011 to 2024. Seventeen surveys meeting inclusion criteria were identified. For each survey, we extracted the proportion of culture-confirmed TB cases that were also identified by symptom screening, chest X-ray (CXR), sputum smear microscopy, and GeneXpert MTB/RIF. We additionally tabulated national HIV prevalence, HIV co-infection rates reported by national TB programs, and HIV co-infection rates documented in the prevalence surveys themselves.

**Results:**

TB prevalence across the 17 surveyed countries ranged from 119 per 100,000 population in Rwanda to 737 per 100,000 in South Africa, with Lesotho (581), Tanzania (590), Zambia (638), Kenya (558), Nigeria (524), Uganda (401), Namibia (465), Malawi (452), Ghana (356), Eswatini (352), Mozambique (334), Zimbabwe (344), Gambia (212), Sudan (183), and Ethiopia (183) recording intermediate burdens. Symptom screening missed an average of 50% of culture-confirmed pulmonary TB cases across all surveys. Smear microscopy missed approximately 60% of bacteriologically confirmed cases. GeneXpert, while more sensitive, still missed an average of 30–35% of culture-confirmed cases even under rigorous study conditions. In contrast, CXR demonstrated concordance exceeding 80% with culture-confirmed TB across almost all surveyed countries. HIV co-infection among TB patients in prevalence surveys was consistently lower than rates reported in national TB programs, suggesting systematic underrepresentation of HIV-positive individuals in survey populations.

**Conclusion:**

Symptoms-based screening and GeneXpert alone are insufficient to detect the majority of people with active, bacteriologically confirmed TB. Chest X-ray, with markedly higher sensitivity than symptom screening or molecular testing under real-world survey conditions, should be repositioned as a primary screening tool in high-burden settings.

## 1 Introduction

Lesotho is a country with one of the highest rates of TB in the world. Despite significant investments in the health system and success of HIV treatment scale-up, TB case finding—using symptoms screening and GeneXpert for diagnosis--has remained low, with the national program finding only 50% of incident cases. Lesotho underwent its first TB prevalence study in 2019 and in reviewing these findings of this study, the authors recognized that even in the setting of a carefully constructed prevalence survey, the standard measures for screening and diagnosis TB did not detect the presence of culture positive pulmonary TB. This review was undertaken to determine if the systematic under detection of culture positive TB by standard methods--systems screening and GeneXpert--were true in other prevalence surveys in Africa.

*Mycobacterium tuberculosis* (TB) is a significant global health issue, with an estimated 10.8 million new cases and 1.3 million deaths in 2024 (1). TB is both curable and preventable. Yet it remains a leading killer in lower- and middle-income countries (LMICs). The strategy of Directly Observed Therapy-Short Course (DOTS) started in the 1970s, and prioritized the diagnosis and treatment of patients who were most likely infectious. Those with smear-positive, pulmonary TB. Thus, within DOTS programs, the entry point is symptom screening: cough of at least 2 weeks’ duration (with other clinical symptoms added later). Diagnosis within DOTS was limited to sputum analysis by microscopy (2). Even prior to the global HIV pandemic, smear positive pulmonary TB represented less than 70% of all TB cases. Children (3), those with extrapulmonary TB (4, 5), and patients co-infected with HIV are less likely to produce positive sputum samples (6, 7). GeneXpert— a molecular test that simultaneously detects the presence of TB as well as the mutation that confers rifampicin resistance — became available starting in 2011 as a programmatic diagnostic tool (8, 9) and demonstrated superior sensitivity compared to smear microscopy. Yet, despite global scale-up of GeneXpert millions of people with TB remain undiagnosed or untreated, resulting in continued community transmission and preventable deaths. TB incidence rates have declined only marginally, particularly in high-HIV-prevalence settings (10) and TB prevalence remains high creating a large reservoir for ongoing transmission.

Accurately estimating the prevalence of TB is methodologically challenging. National TB programs typically rely on estimated incidence (new cases per year) as a proxy for overall burden. In high-burden settings with weak case detection, the pool of prevalent cases—those with active disease from prior years that has gone undetected or been inadequately treated—constitutes a significant and underappreciated portion of the total burden. To quantify this, the WHO recommends population-based TB prevalence surveys using a cluster sampling methodology (Figure 1), in which randomly selected clusters of up to 1,000 adults are screened with both WHO 4-symptom screening tool and chest X-ray and using sputum culture as the gold standard for case confirmation (11, 12).

**Figure 1:**
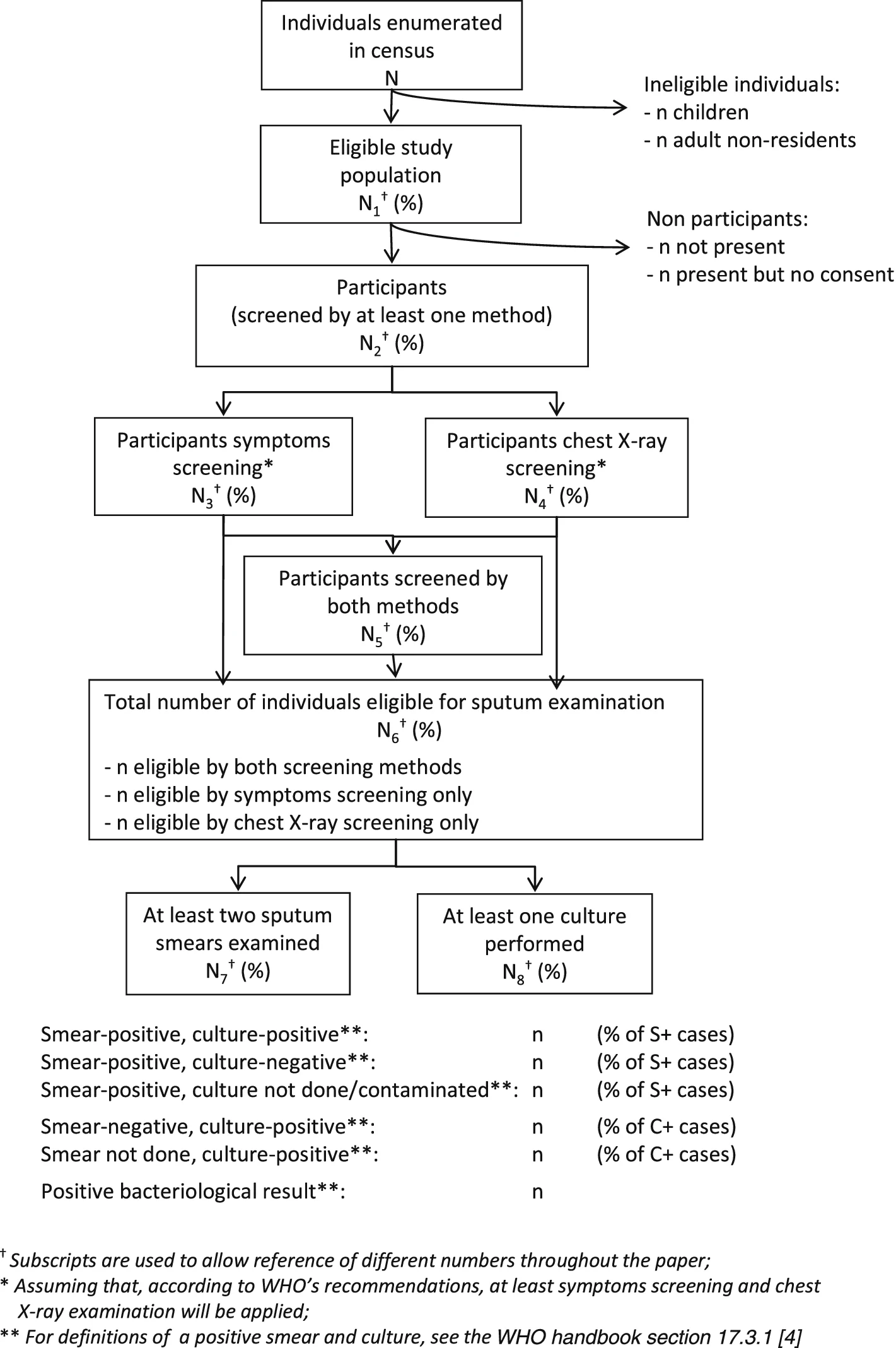
Outline of a World Health Organization (WHO) recommended TB prevalence survey methodology. Cluster sampling of adults aged ≥15 years residing in randomly selected subcommunes. All participants undergo symptom screening and chest X-ray; those with a positive symptom screen or CXR abnormality undergo sputum examination by smear microscopy or GeneXpert and culture. Culture positivity is the gold standard for TB case confirmation (12).

Our review of prevalence studies was prompted by the results of the Lesotho TB prevalence study which demonstrated that in a well sampled adult population among the prevalent cases of culture positive pulmonary TB 70% of patients did not have symptoms and 32% had a negative GeneXpert test. We undertook this study to understand if current methods of screening (symptoms as a point of entry to the TB program) and diagnosis (GeneXpert) were systematically underestimating cases of TB not only in Lesotho but among the 17 TB prevalence surveys conducted in Africa between 2011 and 2024. Based on these findings, we propose a revised programmatic algorithm for TB case finding currently being piloted in Lesotho, a country with one of the highest TB and HIV prevalences in the world.

## 2 Methods

### 2.1 Identification of surveys

A literature search was conducted using PubMed and Google Scholar with the terms “tuberculosis,” “prevalence,” “survey,” and “Africa” for publications from 2011 to 2024. Of 175 papers identified, 17 were included in the final review. Exclusion criteria were multi-drug-resistant TB prevalence surveys; surveys of bovine tuberculosis; surveys restricted to specific occupational groups (miners, health workers); and surveys that did not include both symptomatic screening and chest X-ray as part of the screening protocol.

The 17 surveys included were conducted in Ethiopia (2010–2011), Gambia (2011–2013), Ghana (2013), Kenya (2015–2016), Malawi (2013–2014), Nigeria (2012), Rwanda (2012), Sudan (2013–2014), Tanzania (2011–2012), Uganda (2014–2015), Zambia (2013–2014), Zimbabwe (2014), Eswatini (2018), Mozambique (2019), South Africa (2018), Namibia (2019), and Lesotho (2019).

### 2.2 Survey methodology

In each survey, the eligible population comprised adults aged ≥15 years residing in a household within the sample cluster area for more than 2 weeks. Participants were screened using both a standard WHO 4-symptom screening tool for TB (cough, fever, weight loss, night sweats) and chest X-ray. The primary symptom screening criterion was a cough lasting ≥2 weeks; in some countries, the algorithm was broadened to include cough of any duration and constitutional symptoms (weight loss, chest pain, night sweats, and fever). Participants screening negative on both interview and CXR were considered ineligible for sputum examination and assumed not to have active TB.

Individuals with a positive symptom screen or CXR findings suggestive of TB were referred for sputum examination, including smear microscopy (replaced by GeneXpert in later surveys) and culture for *Mycobacterium tuberculosis*. A positive culture result was considered the definitive (gold standard) diagnosis of TB.

### 2.3 Data extraction and analysis

Results from each survey were extracted and summarised in a standardized table (Table 1), recording the number of bacteriologically (culture) confirmed pulmonary TB cases and the proportion of those cases who also had: (a) a positive symptom screen; (b) CXR suggestive of TB; (c) positive smear microscopy or GeneXpert. Table 2 presents the relationship between national HIV prevalence, HIV prevalence among TB patients in national programs, and HIV co-infection rates reported in the prevalence surveys. For HIV data, we used the 2021 World Bank country estimates (13) and the 2022 WHO Global TB Report.

**Table 1:** TB prevalence surveys, African countries: 2011–2019.

Country	Year	Estimated Prevalence per 100,000 pop	MTB Culture Positive	% X-ray positive	% Symptom positive	% Smear positive	% GeneXpert positive
Lesotho	2019	581	100%	98%	36%	Not done	67%
Mozambique	2019	334	100%	Not done	54%	Not done	Not reported
Namibia	2018	465	100%	99%	49%	Not done	62%
Eswatini	2018	352	100%	Not done	39%	Not done	Not reported
South Africa	2018	737	94%	93%	42%	Not done	65%
Kenya	2015	558	100%	88%	59%	46%	77%
Uganda	2015	401	100%	90%	49%	41%	Not done
Zambia	2014	638	87%	83%	61%	51%	Not done
Zimbabwe	2014	344	100%	87%	36%	22%	Not done
Malawi	2013	452	100%	49%	70%	47%	Not done
Ghana	2013	356	100%	75%	41%	32%	Not done
Sudan	2013	183	100%	79%	46%	51%	Not done
Nigeria	2012	524	100%	73%	64%	70%	Not done
Gambia	2012	212	92%	84%	61%	36%	Not done
Rwanda	2012	119	100%	90%	48%	68%	Not done
Tanzania	2012	590	100%	72%	63%	84%	Not done
Ethiopia	2011	183	97%	90%	52%	43%	Not done

**Table 2:** HIV Prevalence from UNAIDS 2022 and HIV prevalence in national TB programs 2022 vs. HIV prevalence in TB prevalence surveys.

Country	Baseline HIV prevalence adults 15–49, nationally and TB program		Data of HIV positive persons in prevalence survey		
	HIV prevalence (40) 2022	HIV prevalence in TB program (41) 2022	Year	TB Prevalence per 100,000	HIV prevalence among TB patients in the study
Lesotho	21%	59%	2019	581	30%
Mozambique	13% (42)	23%	2019	334	a
South Africa	18%	71%	2018	737	28%
Eswatini	28%	56%	2018	352	a
Namibia	12%	30%	2018	465	a
Kenya	4%	25%	2015	558	17%
Uganda	5%	33%	2015	401	27%
Zambia	11%	39%	2014	638	27%
Zimbabwe	12%	54%	2014	344	51%
Malawi	7%	45%	2013	452	28%
Ghana	2%	18% (43)	2013	356	a
Sudan	0%	14% (44)	2013	183	a
Nigeria	1%	26% (45)	2012	524	a
Gambia	2%	16% (46)	2012	212	a
Rwanda	2%	19%	2012	119	3%
Tanzania	5%	21%	2012	590	a
Ethiopia	1%	7%	2011	180	a

a. HIV test was not done as a routine part of the surveillance program.

Comparison of national HIV prevalence (adults), HIV prevalence among TB patients in national programs (WHO Global TB Report 2022), and HIV co-infection rates reported in national TB prevalence surveys. “a” = HIV testing not conducted routinely in this survey.

a = HIV testing not conducted routinely as part of the prevalence survey; − = data not available in published survey report.

### 2.4 Ethical approval

Ethical approval was not required for this study as it is a review of previously published national TB prevalence surveys, each of which received approval from their respective country ethics committees before conduct.

## 3 Results

### 3.1 TB prevalence across surveys

TB prevalence among the 17 African countries had the following results for TB prevalence per 100,000 population Lesotho (2019) 581, Mozambique (2019) 334, Namibia (2018) 465, Eswatini (2018) 352, South Africa (2018) 737, Kenya (2015) 558, Uganda (2015) 401, Zambia (2014) 638, Zimbabwe (2014) 334, Malawi (2013) 452, Ghana (2013) 356, Sudan (2013)183, Nigeria (2012) 524, Gambia (2012) 212, Rwanda (2012) 119, Tanzania (2012) 590, Ethiopia (2011). This variation likely reflects differences in HIV prevalence, health system strength, and population-level risk factors including occupational silica exposure from mining and household overcrowding. Full results are presented in Table 1.

The standard diagnosis was sputum culture for TB. The concordance of each screening and diagnostic test is reported against the presence of a positive culture.

### 3.2 Performance of chest X-ray

Chest X-ray was the most sensitive screening tool across all surveys, with an average concordance of 83% with bacteriologically confirmed TB cases. The concordance was highest in Namibia (99%) (14), Lesotho (98%) (15), and South Africa (93%) (16). The only notable exception was Malawi, where CXR concordance was 49% (17), reflecting differences in CXR reading protocols or film quality. These findings are consistent with TB prevalence surveys conducted in Asia (18).

### 3.3 Performance of symptom screening

The concordance between a positive symptom screen using the standard WHO 4-symptom screening tool (cough, fever, night sweats and weight loss) and bacteriologically confirmed TB was consistently low across surveys. The lowest concordance was recorded in Zimbabwe (36%) (19), Lesotho (36%) (15), Ghana (41%) (20), and South Africa (42%) (16). Slightly higher concordance was observed in Malawi (70%), Nigeria (64%) (21), Tanzania (63%) (22), Zambia (61%) (23), and Gambia and Ethiopia (52%) (17, 24). Countries that used broader symptom screening criteria—including cough of any duration and constitutional symptoms—achieved higher but still inadequate concordance. Even in the best performing surveys, symptom screening alone would have missed at least 30% of bacteriologically confirmed TB cases.

### 3.4 Performance of smear microscopy and GeneXpert

Most countries used smear microscopy in their prevalence surveys, with an average concordance of approximately 50% between smear positivity and culture-confirmed TB. Countries using smear microscopy—Nigeria, Rwanda, Sudan, Zambia, Zimbabwe, Ghana, Kenya, and Uganda—recorded concordance averaging 50% (19–21, 23, 25–28), consistent with the known sensitivity limitations of microscopy, particularly in HIV-co-infected and paucibacillary populations.

In the more recent surveys, GeneXpert replaced smear microscopy. While GeneXpert demonstrated superior performance compared to smear, it still failed to identify 30–35% of culture-confirmed pulmonary TB cases. The average GeneXpert concordance with culture-positive TB in Namibia, South Africa, Lesotho, and Kenya was 62, 65, 67, and 77%, respectively (14–16, 27). This finding is clinically and programmatically significant: GeneXpert is currently the WHO-recommended gold standard for programmatic TB diagnosis, yet even under the rigorous conditions of a national prevalence survey, it missed nearly one-third of culture-confirmed cases.

### 3.5 HIV co-infection in prevalence surveys vs. national programs

Table 2 presents HIV prevalence data at the national level, within national TB programs, and as reported in the prevalence surveys. In countries where HIV testing was routinely conducted as part of the survey, the prevalence of HIV co-infection among survey participants with confirmed TB was consistently lower than that reported in national TB programs. For example, in Lesotho, where 59% of TB patients in the national program are HIV-positive, only 30% of TB patients in the 2019 prevalence survey were HIV-positive. Similarly, in South Africa (71% vs. 28%), Zimbabwe (54% vs. 51%), and Uganda (33% vs. 27%). This discrepancy reflects a systematic under-representation of people living with HIV (PLHIV) in prevalence surveys, as HIV-positive individuals with TB are more likely to have paucibacillary, smear-negative, or extrapulmonary disease—forms of TB less likely to be detected and confirmed by culture under survey conditions. This suggests that the true burden of TB in high-HIV-prevalence countries is underestimated by prevalence surveys.

## 4 Discussion

TB prevalence in Africa remains high despite years of HIV scale up and health systems strengthening. This review of the national TB prevalence surveys conducted in Africa from 2011 to 2024 reveal a consistent and troubling pattern, Even under rigorous research conditions of a prevalence survey, the tools currently used as the primary entry point for the TB program--symptom screening and the tool used as the primary diagnostic test--GeneXpert miss a substantial proportion of people with bacteriologically confirmed active TB. This gap may be driven by the high prevalence of people living with HIV among TB patients who are less likely to have classic symptoms and more often have paucibacillary disease. The low performance of screening and diagnostic tools in the prevalence studies has profound implications for individual patient outcomes and community-level transmission.

### 4.1 Symptom screening as a primary entry point

Symptom screening remains the first step in the TB care cascade in most LMICs. Yet these surveys demonstrate that, on average, symptom screening misses 40–60% of culture-confirmed TB cases (18). The countries that broadened their screening criteria beyond the standard “cough of two weeks”—incorporating constitutional symptoms and any-duration cough—achieved higher but still clinically inadequate concordance. Even in the most sensitive scenarios, 30% of culture-confirmed pulmonary TB would have been missed if symptom screening was the sole entry criterion. These findings call into question the continued use of symptom-only screening as the primary gateway into TB diagnosis and treatment.

### 4.2 The diagnostic sensitivity gap of GeneXpert

More surprising is the magnitude of GeneXpert’s failure to detect culture-confirmed TB even under controlled survey conditions. GeneXpert is now the WHO-endorsed standard for programmatic TB diagnosis, yet it missed 30–35% of culture-confirmed cases across the surveys in which it was deployed. This reflects well-documented biological limitations: GeneXpert requires a minimum bacterial load in the sputum specimen, and many patients—particularly those co-infected with HIV, malnourished, or in early diseases, have paucibacillary infections with bacillary burdens below the detection threshold (5, 29). In high-HIV-prevalence countries such as Lesotho, Eswatini, South Africa, and Namibia, where extrapulmonary and smear-negative TB forms are disproportionately common, the programmatic sensitivity of GeneXpert is even lower than the survey estimates suggest. Countries should invest in expanding culture and drug susceptibility testing (DST) capacity to address these diagnostic gaps, in line with the WHO End TB Strategy.

### 4.3 Chest X-ray as the Most sensitive screening tool

The most consistent finding across all 17 surveys was the high concordance between CXR abnormality and bacteriologically confirmed TB, averaging 83% across all surveys and reaching 98–99% in Lesotho and Namibia. These findings align with data from TB prevalence surveys conducted in Asia (18, 30, 31). While CXR is not specific for TB, its sensitivity markedly exceeds that of symptom screening or GeneXpert alone. Used as a screening (rather than diagnostic) tool—to identify individuals requiring further workup rather than to confirm the diagnosis—CXR combined with GeneXpert creates a complementary algorithm that reduces the proportion of TB cases missed.

A critical enabler of scaled CXR screening in low-resource settings is artificial intelligence-based computer-aided detection (AI-CAD). AI-CAD tools including CAD4TB, qXR, and Lunit INSIGHT CXR-TB have been validated in sub-Saharan African high-burden settings and achieve sensitivities of 86–91% for TB detection, performing at or above the level of general physicians in CXR interpretation (32–34). AI-CAD eliminates the requirement for on-site radiologists, reduces inter-reader variability, provides immediate results at the point of care, and can operate on local servers without internet connectivity. The integration of AI-CAD with digital CXR removes the primary operational barrier to scaled X-ray screening in LMICs (35).

### 4.4 HIV co-infection and the underestimate of true TB burden in prevalence studies based on culture positive, pulmonary TB

The discrepancy between HIV co-infection rates in national programs versus prevalence surveys (Table 2) points to a structural limitation of all prevalence surveys: their reliance on ambulatory adults able to attend screening sites and produce sputum samples. People living with HIV, who have higher rates of paucibacillary pulmonary TB as well as extrapulmonary TB, are systematically under-captured by survey methodology. This means that the already alarming prevalence figures from these surveys are, in fact, underestimates of the true burden—particularly in countries with high HIV prevalence such as Lesotho (17% HIV prevalence among adults aged 15–49), South Africa, Eswatini, and Zambia. Similarly, children, individuals with extrapulmonary TB, and those in congregate settings (prisons, mining camps) are excluded from standard prevalence survey methodology by design.

### 4.5 Limitations

This review has several limitations. The 17 surveys were conducted over a 13-year period with heterogeneous protocols, CXR reading methods, and sputum collection procedures. Not all surveys reported complete data for all screening and diagnostic modalities, resulting in missing values for some comparisons. All surveys were completed before the COVID-19 pandemic, which is known to have severely disrupted TB services globally (36, 37). The representativeness of survey populations—which exclude children, the hospitalized, and those in congregate settings—limits the generalizability of findings to the full burden of TB. Additionally, the lower HIV co-infection rates in survey populations compared with national program reports suggest that the surveys underestimate TB burden in high-HIV settings.

### 4.6 A way forward: the Lesotho revised algorithm

The study was undertaken by clinician-researchers at the non-profit Partners In Health and Harvard University with the TB program of Ministry of Health in Lesotho understand if the patterns we saw in the Lesotho prevalence study, nearly 64% of those with culture proven pulmonary TB had no symptoms and 36% of those with culture proven pulmonary TB had negative GeneXpert. We sought to use the evidence generated from this study to posit a new way forward for screening and diagnosis of TB in Lesotho and beyond.

Because of the low concordance between symptoms and culture positive TB both in the Lesotho prevalence study (36% of people with positive culture had symptoms) and the prevalence studies reviewed here (an average 50% of people with positive culture in the studies reviewed had symptoms), we proposed a new screening algorithm to replace the reliance on symptoms alone to refer patients to a diagnostic test (Figures 2, 3). The revised algorithm replaces the symptom-only entry point with a two-stage approach: (1) epidemiologic risk screening, which identifies individuals at elevated risk on the basis of age (≥45 years), HIV-positive status, household contact with a known TB case, occupation (mining), or underlying conditions (diabetes, malnutrition); and (2) CXR with AI-CAD for all individuals meeting any risk criterion or reporting any respiratory symptom, with decentralised GeneXpert confirmation for those with CXR findings suggestive of TB. Early programmatic results from the Lesotho pilot are promising and will be reported in subsequent publications.

**Figure 2:**
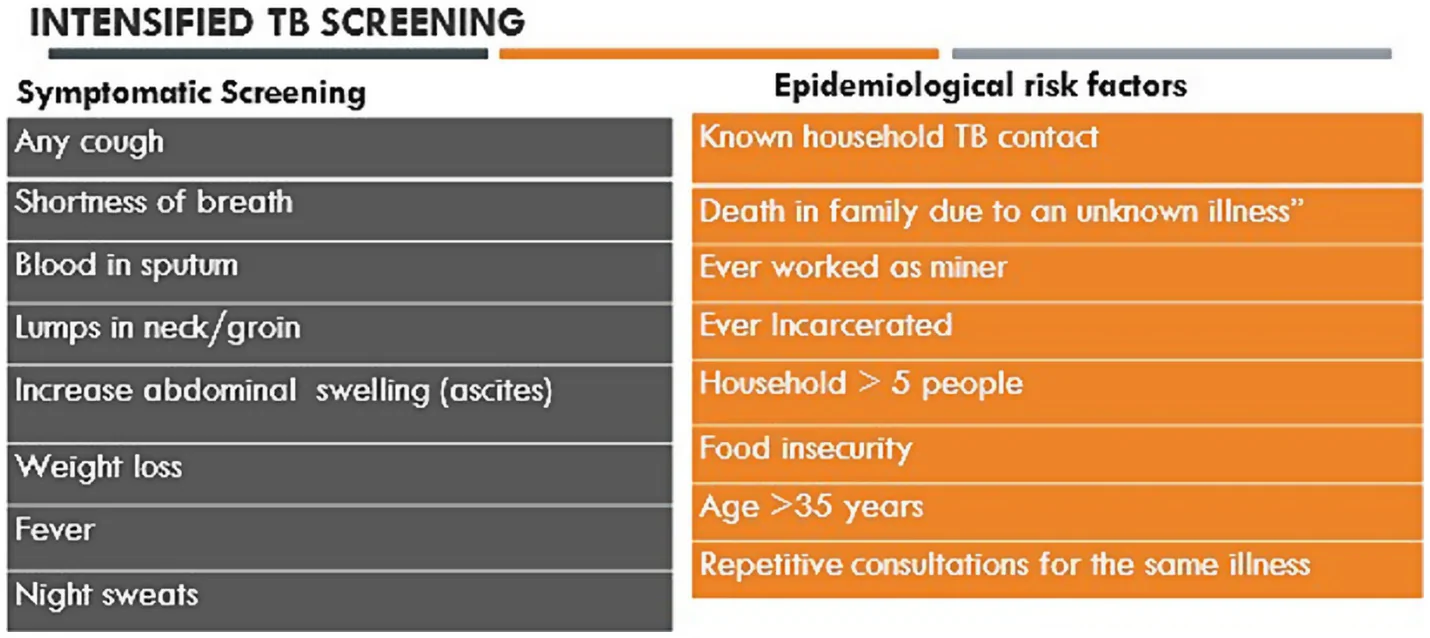
Revised algorithm for the use of expanded epidemiologic screening and chest X-ray in TB case finding, currently being piloted in Lesotho by Partners In Health and the Ministry of Health. The algorithm begins with risk-factor-based epidemiologic screening, followed by digital chest X-ray with AI-CAD for those meeting risk criteria or with any respiratory symptom, and GeneXpert confirmation at the health centre level for all screen-positive individuals.

**Figure 3:**
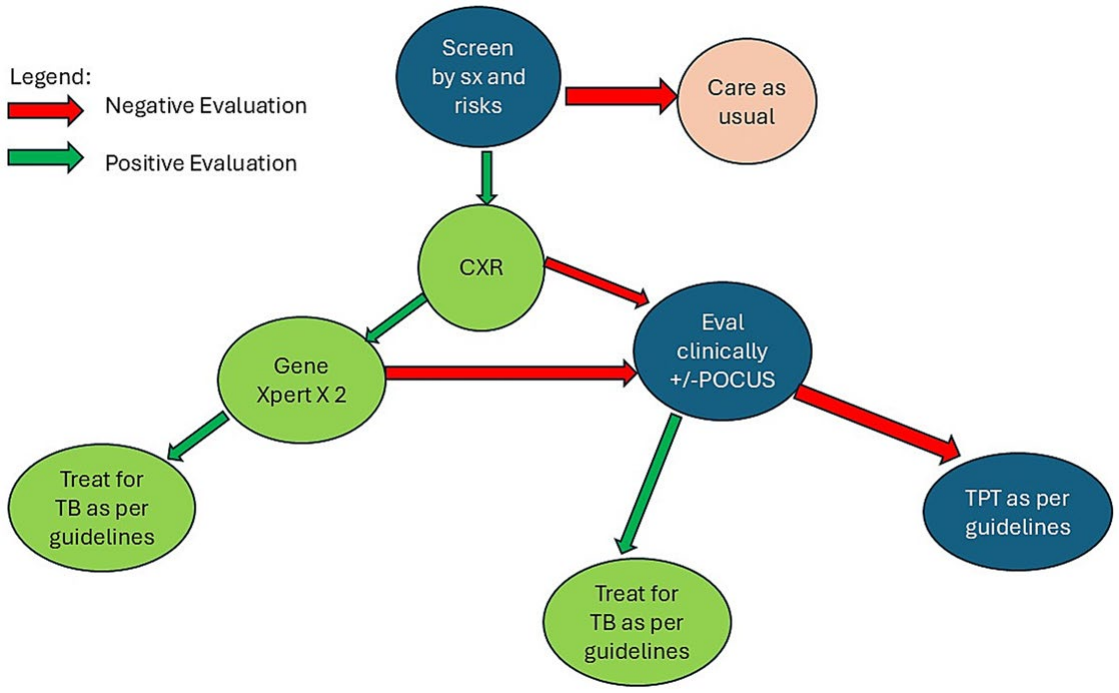
Expanded epidemiologic screening criteria used in the revised Lesotho TB algorithm. Screening identifies individuals at elevated TB risk based on age (≥45 years), HIV status, household contact with a known TB case, occupational exposure (mining), or comorbid conditions (diabetes, malnutrition). All screen-positive individuals proceed to Chest X-ray with AI-CAD regardless of symptom status.

This approach is supported by international evidence. Vietnam’s Double X (2X) strategy, which combines CXR and GeneXpert for systematic TB case finding in community and facility settings, achieved TB detection yields of 19 CXRs per diagnosed case in high-risk populations and demonstrated that programmatic integration of AI-CAD improves CXR sensitivity and reduces inappropriate GeneXpert referrals [Innes et al., (38)]. A cluster-randomized controlled trial in Ca Mau Province, Vietnam further demonstrated that 3 years of annual community-wide active screening reduced the prevalence of microbiologically confirmed TB by 44% compared to passive case detection [Marks et al. (39), NEJM, 2019].

## 5 Conclusion

The national TB prevalence surveys conducted in Africa from 2011 to 2024 provide rigorous, population-level evidence that the current standard approaches to TB case finding—symptom screening as the entry point and GeneXpert as the primary diagnostic test—are insufficient to detect the majority of people with active, bacteriologically confirmed TB. Chest X-ray, with an average concordance of 83% with culture-confirmed TB and reaching 98–99% in the highest-burden countries in this dataset, is the most sensitive TB screening tool available and should be repositioned as a primary screening instrument in high-burden settings. Decentralized GeneXpert confirmation at the health center level, integrated into a risk-stratified epidemiologic screening algorithm, offers a viable path to closing the TB case detection gap. Countries with a high TB burden should urgently revise their national TB screening algorithms to incorporate these tools, invest in the enabling infrastructure required to deploy them at scale, and ensure that the gains made in TB treatment and HIV programs are not undermined by continued failure to find the patients who most need care.
